# Author Correction: Energy implications of the 21^st^ century agrarian transition

**DOI:** 10.1038/s41467-021-24752-y

**Published:** 2021-07-16

**Authors:** Lorenzo Rosa, Maria Cristina Rulli, Saleem Ali, Davide Danilo Chiarelli, Jampel Dell’Angelo, Nathaniel D. Mueller, Arnim Scheidel, Giuseppina Siciliano, Paolo D’Odorico

**Affiliations:** 1 Department of Environmental Science, Policy, and Management, University of California, Berkeley, Berkeley, CA USA; 2 Institute of Energy and Process Engineering, ETH Zurich, 8092 Zurich, Switzerland; 3 Department of Civil and Environmental Engineering, Politecnico di Milano, Milan, Italy; 4 Department of Geography and Spatial Sciences, University of Delaware, Newark, DE USA; 5 Sustainable Minerals Institute, University of Queensland, St Lucia, Australia; 6 Institute for Environmental Studies (IVM), Vrije Univeristeit Amsterdam, Amsterdam, The Netherlands; 7 Department of Ecosystem Science and Sustainability, Colorado State University, Fort Collins, CO USA; 8 Department of Soil and Crop Sciences, Colorado State University, Fort Collins, CO USA; 9 Institut de Ciència i Tecnologia Ambientals (ICTA-UAB), Universitat Autònoma de Barcelona, Bellaterra, Spain; 10 Centre for Development, Environment and Policy, SOAS, University of London, London, UK

**Keywords:** Environmental impact, Sustainability

Correction to: *Nature Communications* 10.1038/s41467-021-22581-7, published online 19 April 2021.

The original version of this Article contained an error in the caption of Fig. 2, where the sentence ‘Yield-gap fractions close to zero show low-yielding croplands with high yield gaps.’ should have been deleted. This has been corrected in both the PDF and HTML versions of the Article.

