# Supplementary material for: Energy implications of the 21st century agrarian transition
Source: Nat Commun. 2021 Apr 19;12:2319. doi: 10.1038/s41467-021-22581-7 (PMC8055646; doi:10.1038/s41467-021-22581-7)
Supplement: Supplementary file 1 — Supplementary Information. [file 41467_2021_22581_MOESM1_ESM.pdf]

Supplementary Materials for:

Energy implications of the 21st century agrarian transition

Authors: Lorenzo Rosa[1,2], Maria Cristina Rulli[3], Saleem Ali[4,5*], Davide Danilo Chiarelli[3], Jampel Dell'Angelo[6], Nathaniel D. Mueller[7,8], Arnim Scheidel[9], Giuseppina Siciliano[10], Paolo D'Odorico[1]

Affiliations:

[1] Department of Environmental Science, Policy, and Management, University of California, Berkeley, United States of America;

[2] Institute of Energy and Process Engineering, ETH Zurich, 8092 Zurich, Switzerland;

[3] Department of Civil and Environmental Engineering, Politecnico di Milano, Milan, Italy;

[4] Department of Geography and Spatial Sciences, University of Delaware, Newark, Delaware, USA;

[5] Sustainable Minerals Institute, University of Queensland, Australia

[6] Institute for Environmental Studies (IVM), Vrije Univeristeit Amsterdam, Amsterdam, The Netherlands;

[7] Department of Ecosystem Science and Sustainability, Colorado State University, Fort Collins, CO, USA;

[8] Department of Soil and Crop Sciences, Colorado State University, Fort Collins, CO, USA;

[9] Institut de Ciència i Tecnologia Ambientals (ICTA-UAB), Universitat Autònoma de Barcelona, Spain;

[10] Center for Development Environment and Policy, SOAS, University of London, UK;

* **Corresponding Author** - Email address: saleem@alum.mit.edu

Data Acquisitions Statement

All of the figures are from publicly available data sets or the calculation basis and assumptions along with the references have been provided. This supplementary materials section further elucidates the data analytical methods as well.

Energy usage for irrigation on land deals

Irrigation energy is a function of the volume of irrigation water and of the hydraulic head. Specifically, the volume of irrigation water is mainly governed by climate, soil type and crop type, and by water losses associated with leaks and the limited efficiency of the irrigation system. The hydraulic head depends on the distance between the water withdrawal source and the cropland. Moreover, the hydraulic head is also influenced by the operating pressure of the irrigation system. The energy input (E) required for each land deal has been assessed considering

surface and sprinkler irrigation systems. Specifically, the energy requirement has been calculated using the following equation:

$$E = \frac{V * TH}{\mu_{PUMP} * \mu_{MOTOR}}$$

where V is the volume of water withdrawal from the water body, TH is the total pressure head (or pumping head), μ_{PUMP} and μ_{MOTOR} represent the pump and the motor efficiency, respectively. A value of 0.8 (80%) and 0.65 (65%) has been used for pump and motor efficiency, respectively[1]. The volume of irrigation water has been assessed using the spatially distributed crop water model WATNEEDS[2] and considering for each deal the cultivation of the specific crops as reported by the Land Matrix. The total pressure head (*TH*) has been calculated according to the type of irrigation system, as described below.

Surface irrigation system calculations

Because land deals are situated on very uneven topography, we considered an average hydraulic head equal to 3 meters representing the water lift from the river/channel where water is withdrawn to the place where it is used. In the case of water withdrawal located far away from the field, we considered an additional lift calculated as the product between the average slope of the channel delivering water to the field (assumed to be around 0.001) and the distance to the field.

Sprinkler irrigation system calculations

The total head (*TH*) required for the sprinkler system was calculated as the sum of the groundwater depth, the operational pressure assumed to be equal to 3 bar (30.59m of water column), the distributed friction losses of the suction/delivery pipelines, the distributed friction losses and concentrated losses along each lateral. Groundwater table depth was evaluated in the centroid of each deal considering Fan et al. (2013) (ref. 3) and distributed and localized losses for the pump and the laterals was investigated for an average square field of 200ha, having 14 laterals with 14 sprinklers each, 100 meters apart. In this configuration, each sprinkler irrigates an area of approximately 0.15ha in 8 hours. The pump friction losses were calculated as the head losses in a reference pipe of the same length as the required hydraulic head with a nominal diameter of 1.2m and a Hazen-William roughness factor of 80. For the lateral friction losses, distributed friction losses were estimated using the Hazen-William formulation, where the flow is equal to 1/14 of the total discharge needed to irrigate a 200ha field and the localized losses were assumed to be equal to 7% of distributed losses. This analysis considers provision of

irrigation water where and when needed, regardless of whether land deals are currently irrigated and/or irrigation systems are already in operation.

Supplementary Table 1. Crop-specific values and sources of fossil-fuel based energy footprint in low-input and high-input agriculture.

	GJ/ha/yr			N application in high-input farming (kg/ha)	Reference
	Low Input	High Input	Oil palm and Jatropha mill		
GROUNDNUT	7.88	45.81		33	4
COTTON	20.75	34.44		211	5
RICE	4.33	34.40		150	4
SUGAR BEET	8.20	33.90		160	6
CORN	4.11	27.37		153	4
POTATO	8.60	25.70		167	6
RAPESEED	3.20	24.40		222	6
SUNFLOWER	5.60	22.80		214	6
SORGHUM	0.86	22.48		78	4
CASSAVA	2.50	19.00		212	6
WHEAT	7.74	17.99		68	4
JATHROPA	2.72	17.86	29.14	168	7, 8
RUBBER TREE	4.56	16.00		61	9
OIL PALM	2.20	16.00	35.74	53	6, 10
SOYBEAN	6.37	12.61		4	4
PULSES	3.40	11.46		14	4
HAY	2.00	10.46		7	4
SUGARCANE	4.80	9.40		73	6
BARLEY	2.83	8.91		78	4
EUCALYPTUS	3.24	5.17		40	11

Supplementary References

1. Daccache, A., Ciurana, J. S., Diaz, J. R., & Knox, J. W. (2014). Water and energy footprint of irrigated agriculture in the Mediterranean region. *Environmental Research Letters*, *9*(12), 124014.
2. Chiarelli, D.D., Passera, C., Rosa, L., Davis, K.F., D'Odorico, P. and Rulli, M.C., 2020. The green and blue crop water requirement WATNEEDS model and its global gridded outputs. *Scientific Data*, *7*(1), pp.1-9.
3. Fan, Y., Li, H., & Miguez-Macho, G. (2013). Global patterns of groundwater table depth. *Science*, *339*(6122), 940-943.
4. Pimentel, D. and Pimentel, M.H. eds., 2007. *Food, energy, and society*. CRC press. London.

5. Ozturk, H.H., 2006. An input-output energy analysis in field crop production in Southeastern Anatolia Region of Turkey. *J. Sustain. Agric*, *25*(1).
6. Brehmer, B., 2008. *Chemical biorefinery perspectives: the valorisation of functionalised chemicals from biomass resources compared to the conventional fossil fuel production route*.
7. Santos, O.N.A., Folegatti, M.V., Lena, B.P., Diotto, A.V., Francisco, J.P. and Romanelli, T.L., 2018. Energy analysis of Jatropha curcas under irrigation and rainfed at the Southeast Brazilian humid subtropical. *Agricultural Engineering International: CIGR Journal*, *20*(3), pp.116-126.
8. Prueksakorn, K., Gheewala, S.H., Malakul, P. and Bonnet, S., 2010. Energy analysis of Jatropha plantation systems for biodiesel production in Thailand. Energy for Sustainable Development, 14(1), pp.1-5.; Prueksakorn, K. and Gheewala, S.H., 2008. Full chain energy analysis of biodiesel from Jatropha curcas L. in Thailand. Environmental science & technology, 42(9), pp.3388-3393.
9. Zulekipli, H.A. and Pebrian, D.E., 2019, September. Analysis of Energy Consumption in Rubber Cultivation in Malaysia: A Case Study. In *IOP Conference Series: Earth and Environmental Science* (Vol. 327, No. 1, p. 012004). IOP Publishing.
10. Andarani, P., Nugraha, W.D. and Wieddya, 2017, March. Energy balances and greenhouse gas emissions of crude palm oil production system in Indonesia (Case study: Mill P, PT X, Sumatera Island). In *AIP Conference Proceedings* (Vol. 1823, No. 1, p. 020064). AIP Publishing LLC.;
11. Romanelli, T.L. and Milan, M., 2010. Energy performance of a production system of eucalyptus. *Revista Brasileira de Engenharia Agrícola e Ambiental*, *14*(8), pp.896-903.
